# Proceedings of the North American Pomological Convention

**Published:** 1850-03

**Authors:** 


### Part 2.—Reviews and Notices of New Works.

### ARTICLE I.

*Proceedings of the North American Pomological Convention held at Syracuse, Sept. 14th*, 1849: page 64. 8 vo.

We are indebted to our friend Dr. J. A. KENNICOTT of The Grove, Cook Co., Ills., President of the Convention, for the Pamphlet whose title stands at the head of this article. And what has it to do with Medicine and Surgery? we fancy we hear our readers ask. Can a Convention for promoting the improvement and scientific culture of fruit be of any interest to Medical men? We answer, yes; for fruits are among the most common articles of diet in the great length and breadth of our country, and consequently cannot fail to have an important Hygienic or Morbific influence in preventing, producing and modifying disease. And as their influence to a great extent depends upon their quality, all men are interested in promoting improvements in fruits. And this leads us to remark that the influence of fruits as articles of luxury and diet is too much neglected by our Profession. It has been too much the habit of Physicians to condemn them by wholesale and exclude them altogether from their dietetic prescriptions. So far from being objectionable in the treatment of many diseases, and especially during the period of convalescence, we believe good sound ripe apples, peaches, pears, plumbs, cherries, grapes and other wholesome fruits are often of the greatest utility. They furnish in the most agreeable form for administration vegetable acids, in cases where they are indicated, and prove efficient in regulating the bowels where the administration of directly cathartic remedies is inadmissible. We have found them especially applicable to the fulfilment of these indications in cases of indigestion and constipation.

We have read with much interest several of the reports, but especially that of the Chairman of the Committee for Illinois, by Dr. Kennicott. A copy of this should be in the hands of every farmer, gardener and physician in the country, as it gives many useful suggestions and a great amount of valuable information. The report commences with a description of the topography, soil and climate of Illinois, and treats of their influence on the growth of fruit trees. It next gives a history of the introduction of fruit trees, with statistics of the present state of their cultivation in the State. And afterwards considers the adaptation of different varieties of fruit to our country, and the advantages and difficulties that attend their successful cultivation. It closes with the following just remarks upon "the prophylactic and curative properties of ripe fruits."—

"It has long been known to a few observing men, and now and then a writer has glanced at the fact,—that fruits in season possess remedial virtues. Ripe grapes have cured epidemic dysentery. In vine countries, they speak familiarly of the "grape cure." Physicians have occasionally ventured to recommend the use of "cooling acid fruits;" and the earliest writers have directed the sugary ones, as "figs," for food in convalescence. But it is known to all that many are prejudiced against fruits, and consider them as very questionable *luxuries* at the best. And it must be admitted that they have often proved mischievous, *especially when immature*, and taken by stealth, or in too large quantities, when but occasionally accessible.

Thus, in 99 cases out of every hundred, it will be found that the *abuse*, and not the free use of fruits, has produced the mischief. Good fruits are always grateful,----even to the sickly or palled appetite--and in the young, and the healthy, its promising appearance, or its delicious aroma, often excites the most ungovernable appetite, and they gorge themselves---and suffer therefrom—no worse than from a surfeit of fish, flesh, or vegetables, perhaps—but still enough to aid in perpetuating the vulgar error, that the unrestricted use of fruits is dangerous. Who ever heard of the children of men who provide seasonable fruits in abundance,—and permit their habitual use,—eating too much? or becoming sick therefrom? I never did. I have had a little experience in this matter, and I have taken pains to collect information, and I *know* that the families where fruit is most plentiful, and good, and most highly prized, as an article of DAILY FOOD---are most free from disease of all kinds, and more especially from *fevers* and "*bowel complaints*."

I have theories to account for this---but I love not theorizing---and will not inflict my crude notions on you at this time---I merely state the grave facts, and defy contradiction. I may add, however, that most fruits aid digestion---some directly---some indirectly by restraining the appetite for more gross and stimulating food and condiments---by keeping the "bowels soluble"---in other words acting as mild "laxatives," &c., &c. The juicy ones act as " dilutants, " and all as "*diuretics*"---the free acids neutralizing or rendering soluable the earthy matters in the blood, and carrying them off rapidly through the natural channels.

All this you can understand and appreciate. But let us glance at another phase of this universal remedy. It is the best, the cheapest, and the least exceptionable cure for INTEMPERANCE. It not only lessens the desire for alcoholic drinks, but supplies their place, and removes their effects.

EVE was tempted by an "APPLE." A good God has given us the object of "the primal sin," as a great blessing. If disease and death came from the eating of "the forbidden fruit"---health and length of days may be found in the assiduous cultivation and regulated enjoyment of that from which the interdict of the Creator has been taken, and which His open hand has lavishly scattered over the face of this fair earth." E.

